# Angiomodulin, a marker of cancer vasculature, is upregulated by vascular endothelial growth factor and increases vascular permeability as a ligand of integrin *α*v*β*3

**DOI:** 10.1002/cam4.216

**Published:** 2014-04-16

**Authors:** Eriko Komiya, Hiroki Sato, Naoko Watanabe, Marii Ise, Shouichi Higashi, Yohei Miyagi, Kaoru Miyazaki

**Affiliations:** 1 Department of Genome Science, Graduate School of Integrated Science and Nanobioscience, Yokohama City University641-12 Maioka-cho, Totsuka-ku, Yokohama, Kanagawa, 244-0813, Japan; 2 Division of Cell Biology, Kihara Institute for Biological Research, Yokohama City University641-12 Maioka-cho, Totsuka-ku, Yokohama, Kanagawa, 244-0813, Japan; 3 Division of Cancer Therapy, Kanagawa Cancer Center Research Institute1-1-2 Nakao, Asahi-ku, Yokohama, Kanagawa, 241-0815, Japan

**Keywords:** Angiomodulin, IGFBP, integrin *α*v*β*3, tumor angiogenesis, vascular permeability

## Abstract

Angiomodulin (AGM) is a member of insulin-like growth factor binding protein (IGFBP) superfamily and often called IGFBP-rP1 or IGFBP-7. AGM was originally identified as a tumor-derived cell adhesion factor, which was highly accumulated in blood vessels of human cancer tissues. AGM is also overexpressed in cancer-associated fibroblasts (CAFs) and activates fibroblasts. However, some studies have shown tumor-suppressing activity of AGM. To understand the roles of AGM in cancer progression, we here investigated the expression of AGM in benign and invasive breast cancers and its functions in cancer vasculature. Immunohistochemical analysis showed that AGM was highly expressed in cancer vasculature even in ductal carcinoma in situ (DCIS) as compared to normal vasculature, while its expression in CAFs was more prominent in invasive carcinomas than DCIS. In vitro analyses showed that AGM was strongly induced by vascular endothelial cell growth factor (VEGF) in vascular endothelial cells. Although AGM stimulated neither the growth nor migration of endothelial cells, it supported efficient adhesion of endothelial cells. Integrin *α*v*β*3 was identified as a novel major receptor for AGM in vascular endothelial cells. AGM retracted endothelial cells by inducing actin stress fibers and loosened their VE-cadherin-mediated intercellular junction. Consequently, AGM increased vascular permeability both in vitro and in vivo. Furthermore, AGM and integrin *α*v*β*3 were highly expressed and colocalized in cancer vasculature. These results suggest that AGM cooperates with VEGF to induce the aberrant functions of cancer vasculature as a ligand of integrin *α*v*β*3.

## Introduction

Complex interaction of tumor cells with surrounding stromal cells is thought to produce special microenvironment that promotes tumor progression 1–3. The vascular system is one of the most important components that constitute such tumor microenvironment 4. Neovascularization, or angiogenesis, is required for supplying nutrients and oxygen to growing tumor cells and for supporting tumor metastasis through the vascular system. It is also known that blood vessels in tumor tissues are structurally and functionally distinct from those in normal tissues 5–7. Such structural and functional changes of blood vessels are induced by cytokines and extracellular matrix (ECM) components, both which are produced by endothelial cells themselves, tumor cells, inflammatory cells, and other stromal cells 8.

Angiomodulin (AGM) is a secretory glycoprotein of about 30 kDa. AGM is alternatively called insulin-like growth factor binding protein (IGFBP)-7 or IGFBP-related protein-1 (IGFBP-rP1). Although AGM is thought to be a member of the IGFBP superfamily, it has little affinity for IGFs and its sequence homology to the IGFBP families (IGFBPs-1 to -6) is as low as about 20% 9–11. AGM was originally identified as tumor-derived cell adhesion factor (TAF) secreted by human bladder carcinoma cells 12 and as prostacyclin-stimulating factor (PSF) from human fibroblasts 13, and its cDNA was cloned as mac25 from normal human leptomeningial cells 14. An early study demonstrated that this protein is highly expressed in blood vessels of cancer tissues, proposing the name “angiomodulin” 15. A recent study has shown that AGM plays a synergistic role with vascular endothelial growth factor (VEGF) in angiogenesis 16. The AGM message or protein has been detected in a wide range of normal tissues and cells such as vascular endothelial cells, smooth muscle cells, fibroblasts, and some types of cancer cells 9,12–14,17. Pathological roles of AGM in cancer are controversial. AGM is highly expressed in some cancer cell lines 18, invading tumor cells in colon cancer 19 and cancer vasculature 15. In acute lymphoblastic leukemia, expression of AGM correlates with worse prognosis of patients 20. We recently found that AGM is overexpressed not only by vascular endothelial cells but also by cancer-associated fibroblasts (CAFs) in human carcinomas of the colon, lung and uterus 21. The AGM expression in fibroblasts, which is induced by TGF-*β* in vitro, seems to activate fibroblasts in cancer stroma. On the other hand, other studies showed that AGM may be a tumor-suppressing protein 9. In breast 22, prostate 23, and lung 24 cancers, reduced expression of AGM is correlated with worse prognosis of patients. Forced expression of exogenous AGM in cancer cells inhibits the tumor cell growth by inducing senescence or apoptosis in culture 24–27 or in xenograft models 24,28,29. In a case of melanoma cells, recombinant AGM protein is able to suppress tumor cell growth both in vivo and in vitro 29,30. Thus, exact roles of AGM in cancer progression remain to be clarified.

Breast cancer is the most common cancer in women and often metastasizes into bones and lymph nodes. Based on the origin and progression stage, breast cancers can be classified into noninvasive carcinoma (or ductal carcinoma in situ; DCIS), invasive lobular carcinoma, invasive ductal carcinoma, and other types of carcinomas. Enhanced angiogenesis in breast carcinomas is closely associated with poor survival of patients 31. To get a clue for understanding the roles of AGM in cancer progression, here we investigated the expression and function of AGM in blood vessels of normal breast tissues and noninvasive and invasive breast cancer tissues, as well as its regulatory factors.

## Materials and Methods

### Materials

Human tissue specimens of 32 breast cancers (five normal mammary glands, six ductal carcinoma in situ (DCIS), and 21 invasive carcinomas including six lobular carcinomas and 15 ductal carcinomas) were provided by Human Cancer Tissue Center of Kanagawa Cancer Research and Information Association (KCRIA), Kanagawa, Japan. Normal tissues were obtained from DCIS tissues which did not contain tumor cells. The study protocol was approved by the Ethical Committees of both KCRIA and Kihara Institute for Biological Research, and performed according to the guidelines of the 1995 declaration of Helsinki. Written informed consent was obtained from each patient. Human epidermal growth factor (EGF), basic fibroblast growth factor (bFGF), VEGF-121 and -165 splicing variants (VEGF-121, VEGF-165), interleukin-6 (IL-6), tumor necrosis factor (TNF-*α*), transforming growth factor-*β* (TGF-*β*) were purchased from Wako (Osaka, Japan). Type IV and type I collagens were purchased from Nitta Corporation (Osaka, Japan), interleukin-8 (IL-8) from Bachem California (Walden, Essex, UK), recombinant integrin *α*v*β*3 from R&D (Minneapolis, MN), and recombinant connective tissue growth factor (CTGF/CCN2) from Wako. RGD peptide (Gly-Arg-Gly-Asp-Ser-Pro) and control RGE peptide (Gly-Arg-Gly-Glu-Ser-Pro) were purchased from Takara (Shiga, Japan). Mouse monoclonal antibody (MoAb) against human AGM/TAF/IGFBP-rP1 (clone 88), which is available from Cosmo-Bio (Tokyo, Japan), was previously reported 15. Mouse MoAb against human laminin-511 (clone 12D) was prepared by immunizing mice with human placental laminin in our laboratory. This MoAb can be used for immunohistochemistry, immunoprecipitation, laminin-511 purification, and immunoblotting under nonreduing conditions. Function-blocking anti-integrin antibodies used were anti-*α*2 (P1E6), anti-*α*3 (P1B5), anti-*α*5 (P1D6), anti-β1 (6S6), and anti-*α*v*β*3 (LM609) MoAbs from Merck (Darmstadt, Germany), and anti-*α*6 MoAb (GoH3) from PharMingen (San Diego, CA). Other anti-integrin antibodies used were anti-β3 MoAb (PM6/13) from Abcam (Cambridge, Cambridgeshire, UK) and anti-*α*v MoAb (M9) from Merck. Other antibodies to human antigens used were anti-*β*-actin MoAb from Sigma-Aldrich (St. Louis, MO), anti-CD68 MoAb from Thermo Scientific (Waltham, MA), anti-VEGF rabbit polyclonal antibody (PoAb) from Santa Cruz (Santa Cruz, CA), FITC-labeled anti-CD31 MoAb from BioLegend (San Diego, CA), rabbit PoAb against von Willebrand factor (vWF) from Dako (Glostrup, Denmark), anti-tubulin MoAb form Merck, rabbit PoAb against *α*-smooth muscle actin (*α*-SMA) from Abcam, and goat PoAb against IGFBP-rP1/AGM from R&D.

### Cell culture

Human umbilical vein endothelial cells (HUVECs) were purchased from Kurabo (Osaka, Japan) and stored in liquid nitrogen. The cells were maintained on type-I collagen-coated dishes in MCDB131 medium (Sigma-Aldrich) supplemented with 10% fetal calf serum (FCS; JRH Bioscience, Lenexa, KS), 10 ng/mL EGF, 5 ng/mL bFGF, 50 *μ*g/mL heparin, 100 U/mL penicillin G, and 100 *μ*g/mL streptomycin sulfate at 37 °C in a humidified atmosphere of 5% CO_2_ and 95% air. HUVECs at the third to fifth passages were used in experiments. In some experiments, the medium from which FCS had been deleted was used as serum-free basal medium for HUVECs.

### Purification of AGM

AGM was purified from the serum-free conditioned medium of the human bladder carcinoma cell line T-24 (EJ-1 strain) by the previously reported method 21. Briefly, the conditioned medium was collected every 2 or 3 days from confluent cultures of T-24 cells. AGM in the conditioned medium was concentrated by heparin affinity chromatography and then purified by immuno-affinity chromatography on a column conjugated with the anti-AGM antibody (clone 88).

### Immunohistochemistry of human cancer tissues

Five-*μ*m serial frozen sections were obtained from (KCRIA) and subjected to immunohistochemistry as described previously 32. The sections were treated with the first antibody, biotin-conjugated second antibody (Vector; CA) and then streptoavidin-conjugated peroxidase (Vector). The resultant immunocomplexes were visualized with 3,3-diaminobenzidine (DAB) using the Histofine kit (Nichirei, Tokyo, Japan). Immunostaining intensity was determined by the Image J software (W. S. Rasband; NIH, Bethesda, MD) from a representative microscopic field (830 × 580 *μ*m) of each section, which contained 10–30% tumor cells in area. To grade the CD68 staining semiquantitatively, the immunostaining intensity was scored in a blind fashion as to the origins of cancer samples and graded to 0 (no or little signal), +1 (weak signals), +2 (intermediate signals), and +3 (strong signals). In Immunofluorescence staining, cy3-labeled anti-mouse-IgG antibody, FITC-labeled anti-rabbit-IgG antibody, or FITC-labeled anti-goat-IgG antibody was used as the second antibody (Vector).

### Induction of AGM expression by cytokines in cultured endothelial cells

To examine AGM expression in cultured endothelial cells in response to cytokines, HUVECs were inoculated at a density of 1.5 × 10^5^ cells per well of 24-well plates and incubated for 3 days. After the incubation, the cultures were washed once with MCDB131 and incubated in the presence and absence of a test cytokine in the serum-free basal medium supplemented with 0.05% FCS and 10 *μ*g/mL transferrin in MCDB131 medium. The resultant conditioned medium was collected and clarified by centrifugation. Proteins in the medium was precipitated with 10% (w/v) trichloroacetic acid (TCA), washed with cold ethanol and dissolved in 0.05 mL of the SDS sample buffer for immunoblotting. Lysates were prepared from the cells remaining on the plates and subjected to SDS-PAGE and immunoblotting as internal loading controls.

### Electrophoretic analyses

Sodium dodecyl sulfate polyacrylamide gel electrophoresis (SDS-PAGE) was performed on 12.5% gels under reducing conditions. Separated proteins were stained with Coomassie Brilliant Blue R-250 (CBB). For analyzing specific proteins, conditioned media or cell lysates were separated on the gels, transferred to PVDF membranes, and detected by immunoblotting using specific antibodies and the ECL detection reagents (GE healthcare, Buckinghamshire, UK).

### Assay of AGM binding to integrin αvβ3 by ELISA

AGM was coated at indicated concentrations on 96-well ELISA plates at 4 °C overnight. The coated plates were washed with Tris-buffered saline containing 0.05% (v/v) Tween-20 (TBST) and blocked with 3% (w/v) bovine serum albumin (BSA) at room temperature for 2 h. After washing with TBST, the plates were incubated with 5 *μ*g/mL of a soluble form of integrin *α*v*β*3 in the presence of 10 mmol/L EDTA or 10 mmol/L MgSO_4_ plus 1.6 mmol/L CaCl_2_ at 4 °C overnight, followed by washing with TBST in the presence of EDTA or MgSO_4_/CaCl_2_. Bound integrin was detected by incubating sequentially with anti-*β*3 integrin antibody (PM6/13), a peroxidase-conjugated second antibody and then a reaction mixture containing *o*-phenylendiamine (OPD) and H_2_O_2_. The color was measured for absorbance at 485 nm.

### Cell adhesion assay

Cell adhesion assay was performed as described previously 15 with some modifications. Briefly, test proteins were coated on 96-well ELISA plates (Costar, Cambridge, MA) and blocked with 1.2% (w/v) BSA. HUVECs were inoculated onto the coated plates at a density of 3 × 10^4^ cells per well containing in the serum-free basal medium and incubated at 37 °C for 2 h. After nonadherent cells were removed, adherent cells were fixed and stained with 0.5% (w/v) crystal violet in 20% methanol. After washing with water, the dye was measured for absorbance at 544 nm.

### Endothelial cell permeability assays in vitro and in vivo

In vitro permeability assay was carried out according to the method of Chen et al. 33. HUVECs were grown to confluence on type I collagen-coated culture inserts with 0.4-*μ*m pores (BD bioscience), treated with or without test samples for 18 h. After the incubation, 15 *μ*L of 5 mg/mL FITC-dextran 40 (Sigma-Aldrich) was added to the upper chamber and incubated at 37 °C for 30 min. Fluorescence intensity of FITC-dextran diffused into the lower chamber was measured with the excitation at 485 nm and the emission at 590 nm. Miles vascular permeability assay was carried out using mice according to the method of Zhang et al. 34. Evans blue dye (200 *μ*L of 0.5% solution in 0.9% NaCl) was injected intravenously into mice. Ten min later, a test sample or PBS as control was injected intradermally into the right and left sides, respectively, of the back skin (50 *μ*L each) or the ears (10 *μ*L each) in each mouse. At 30 min, the animals were euthanized, and the skin area including the entire injection site was removed by punch biopsies. Evans blue dye was extracted from the skin tissues with formamide and measured for absorbance at 595 nm.

### Statistical analysis

Statistical significance was evaluated with the unpaired Student's *t*-test. A *P*-value < 0.05 was considered significant.

## Results

### Expression of AGM in blood vessels of noninvasive and invasive breast cancers

To investigate AGM expression in normal and breast cancer tissues, we analyzed 32 human breast tissue samples from different patients, which were classified into four groups: normal mammary epithelial tissues, noninvasive carcinomas (DCIS), and invasive ductal carcinomas, and invasive lobular carcinomas. Serial frozen sections from each tissue sample were subjected to immunohistochemical staining with the anti-AGM antibody (clone 88) and with antibodies against laminin-511 and von Willebrand factor (vWF) as vascular endothelial cell markers and one against CD68 as a macrophage marker.

**Figure 1:**
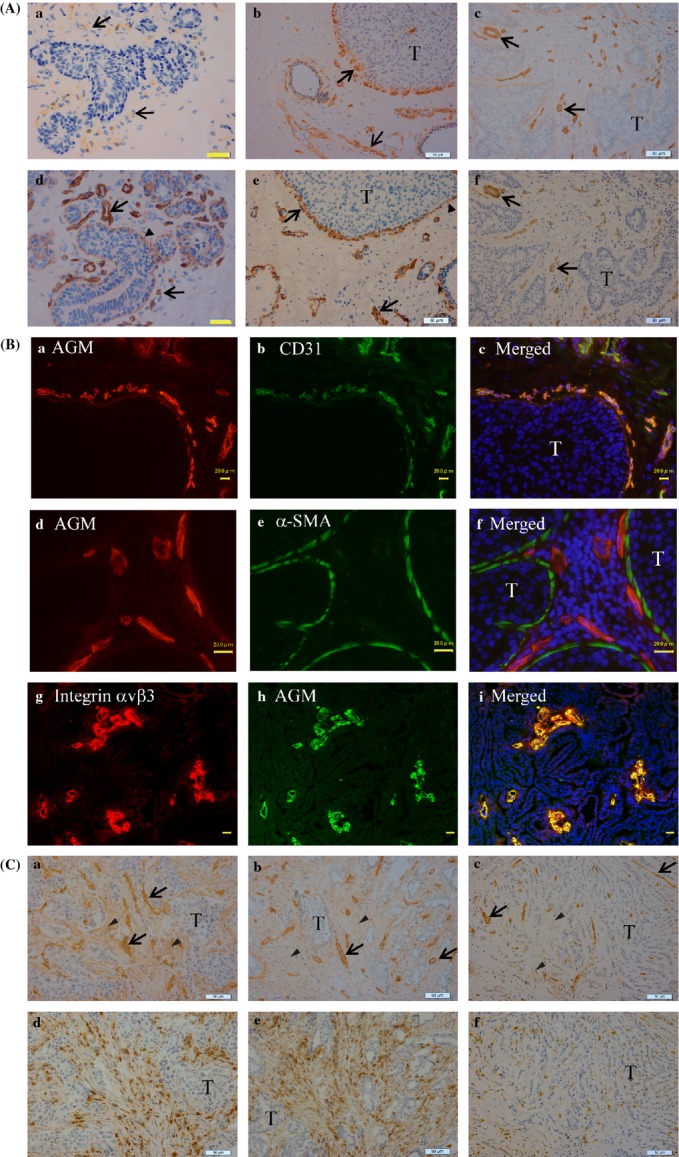
Immunohistochemical analysis of AGM in normal epithelial tissues and cancer tissues of breast. (A) Close sections of normal mammary glands (a and d), DCIS (b and e) and invasive ductal carcinoma (c and f) were immunostained for AGM (a–c) and laminin-511 (d–f) as described in Materials and Methods. Arrows, positive signals in blood vessels; arrowheads, positive signals in basement membranes of normal epithelia or tumor cell islands; T, tumor cells. Yellow scale bars, 20 *μ*m, white scale bars 50 *μ*m. When nonimmune mouse IgG was used as a negative control instead of the first antibody, no staining was found in the same conditions. (B) Double immunofluorescence staining of DCIS (a–f) and invasive carcinoma (g–i) tissues for AGM (a and d, red; h, green) and CD31 (b, green), *α*-SMA (e, green), or integrin *α*v*β*3 (g, red). Scale bars, 20 *μ*m. (C) Immunohistochemical staining of invasive carcinomas for AGM and CD68. Close sections of two invasive ductal carcinomas (a, b, d and e) and one invasive lobular carcinoma (c and f) were immunostained for AGM (a–c) and CD68 (d–f). Arrows, AGM-positive vessles; arrowheads, AGM-positive fibroblasts. Scale bars, 50 *μ*m.

AGM was scarcely or weakly detected in the vasculature of normal breast tissues (Fig. 1A-a), while laminin-511 was highly detected in both vascular and epithelial basement membranes (Fig. 1A-d). Similar immunostaining patterns of AGM and laminin-511 were obtained in normal skin tissues of the breast (data not shown). In DCIS tissues, however, microvessels surrounding tumor cells were prominently stained for both AGM and laminin-511 (Fig. 1A-b and A-e). Laminin-511 was also detected in tumor basement membranes (Fig. 1A-e).

To localize AGM in DCIS and invasive carcinoma tissues more clearly, double immunofluorescence staining with other markers was carried out. The analyses with antibody against the endothelia cell marker CD31 (PECAM) clearly showed that the AGM signals were colocalized with the CD31 signals (Fig. 1B-a–c). Double immunofluorescence staining with anti-*α*-SMA antibody demonstrated that AGM-positive microvessles were located in the close vicinity of tumor cell clusters in DCIS, which were surrounded by *α*-SMA-positive myoepithelial cells (Fig. 1B-d–f). The basement membranes of myoepithelial cells also showed weak immnunoreactivity for AGM. Integrin *α*v*β*3 is often overexpressed in blood vessels of cancer tissues 35. Immunofluorescence staining of invasive carcinomas showed that AGM and integrin *α*v*β*3 were highly overexpressed and colocalized in blood vessels (Fig. 1B-g–i).

In invasive carcinomas, microvessels and venules around carcinoma cells had strong immunosignals for AGM in all cancer tissues tested (Fig. 1A-c, B-h and C-a–c). When compared between ductal carcinomas and lobular carcinomas, the former contained relatively larger venules (Fig. 1C-a and b), while the latter was more abundant with capillaries (Fig. 1C-c). In many of these cancer tissues, the anti-AGM antibody stained blood vessels more clearly than the anti-laminin-511 antibody. A typical case is shown in Figure 1A-c/f.

Our recent study showed that AGM is also expressed by cancer-associated fibroblasts in the lung, colon, and uterus 21. TGF-*β*, which is often expressed by macrophages and tumor cells themselves, was thought to be a major inducer of AGM in the fibroblasts. In the present immunohistochemical study for breast cancers, AGM was clearly seen in the stroma of some invasive carcinomas (Fig. 1C-a and b). Immunostaining for the macrophage marker CD68 showed that these cancer tissues contained a large number of infiltrating macrophages. Macrophages were more abundant in invasive ductal carcinomas than invasive lobular carcinomas (Fig. 1C-d–f).

**Figure 2:**
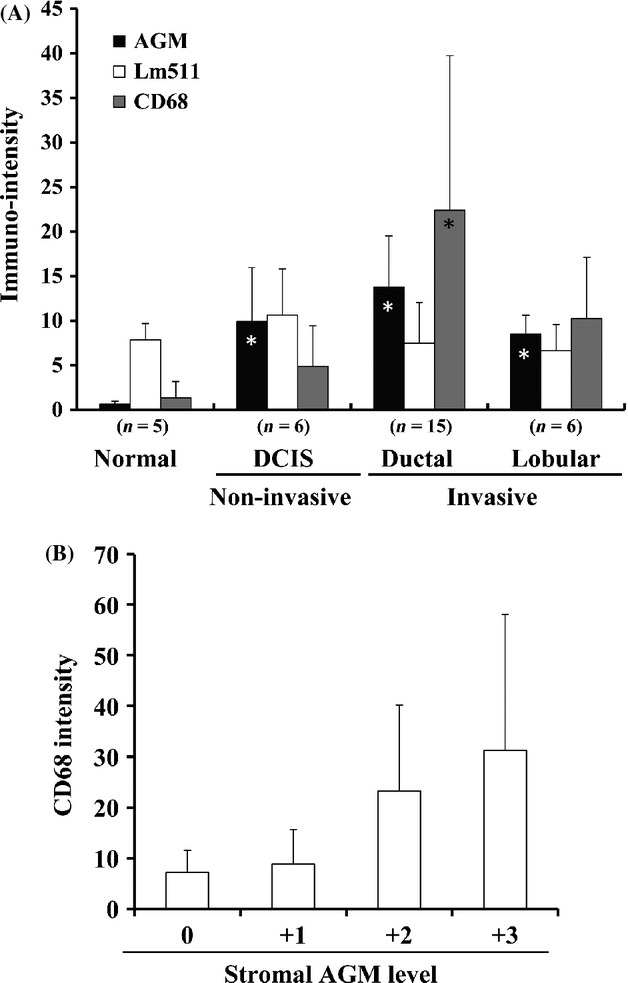
Quantitative analyses of immunohistochemical staining of breast cancer tissues for AGM, laminin-511 and CD68. (A) Thirty-two different tissue specimens were immunostained for AGM, laminin-511 (Lm511) and CD68. Immunostaining intensity was determined by the Image J software from a representative microscopic field (830 × 580 *μ*m) of each section, which contained 10–30% tumor cells or normal epithelial cells in area. Each value represents the average intensity ± SD (bar). The immunopositive signals for laminin-511 in normal and DCIS samples include the signals of epithelial basement membranes. *n*, number of test samples. * statistically significant as compared with normal samples (*P* < 0.01). (B) Relationship between stromal AGM staining and CD68 staining in all samples tested. The stromal AGM staining other than the vascular staining was graded to 0 (no or little signal), +1 (weak signals), +2 (intermediate signals), and +3 (strong signals). In each group of samples, the averaged signal intensity of CD68 staining was determined and plotted in the ordinate. Bars indicate the SD values.

All immunohistochemical data for the AGM, laminin-511 and macrophage expression are summarized in Figure 2A. The total AGM expression in vasculature appeared to be slightly higher in ductal carcinomas than lobular carcinomas and DCIS. The laminin-511 expression was relatively constant in both normal and cancer tissues. Similar results were obtained when anti-vWF antibody was used instead of laminin-511 (data not shown). The quantitative analysis of macrophage staining showed a good correlation between the stromal, but not vascular, expression of AGM and the relative number of tumor-associated macrophages (Fig. 2B). This also suggests that the stromal expression of AGM is related with cancer invasiveness (Fig. 2A).

### Induction of AGM expression by cytokines in cultured vascular endothelial cells

**Figure 3:**
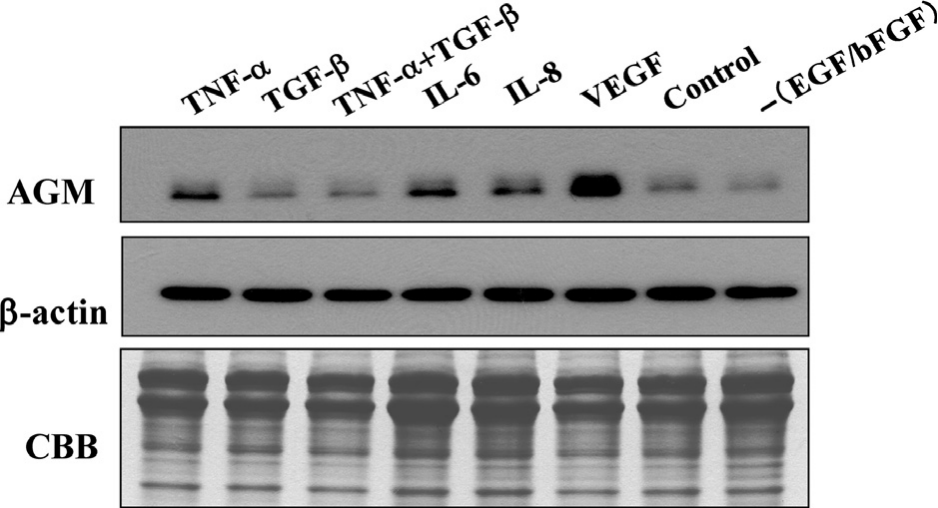
Induction of AGM expression by cytokines in cultured vascular endothelial cells. HUVECs were incubated with the indicated cytokines at the following concentrations in low-serum basal medium for 2 days: 10 ng/mL TNF-*α*, 10 ng/mL TGF-*β*, 100 ng/mL IL-6, 100 ng/mL IL-8, or 50 ng/mL VEGF. After the incubation, conditioned medium and cell lysate were prepared from each culture and used for the detection of AGM and *β*-actin, respectively, by immmunoblotting. The total proteins of the conditioned media were also analyzed by the CBB staining of the gel as internal loading control. – (EGF/FGF), basal medium without EGF and FGF. Other experimental conditions are described in Materials and Methods.

It is expected that the AGM expression in vascular endothelial cells may be regulated by microenvironmental factors secreted by tumor cells and surrounding stromal cells such as endothelial cells, fibroblasts, pericytes, and inflammatory cells. To show the AGM-inducing factors in cancer vasculature, we investigated effects of TNF-*α*, TGF-*β*, VEGF, IL-6, and IL-8 on the expression of AGM protein by HUVECs in vitro (Fig. 3). Expression of AGM was markedly upregulated by VEGF-121 and weakly but significantly by IL-6, IL-8, and TNF-*α*. We could not see any inducing activity of TGF-*β* in HUVECs.

A previous study has suggested the functional interaction between VEGF and AGM 16. Another study has shown that mouse recombinant AGM has affinity for mouse VEGF-164 as assayed by ELISA 36. In this study, we performed similar experiments using human natural AGM and human recombinant VEGF-165 and -121. However, the affinity of AGM for the VEGFs was not evident in our ELISA experiments (data not shown).

### Cell adhesion to AGM via integrin αvβ3

**Figure 4:**
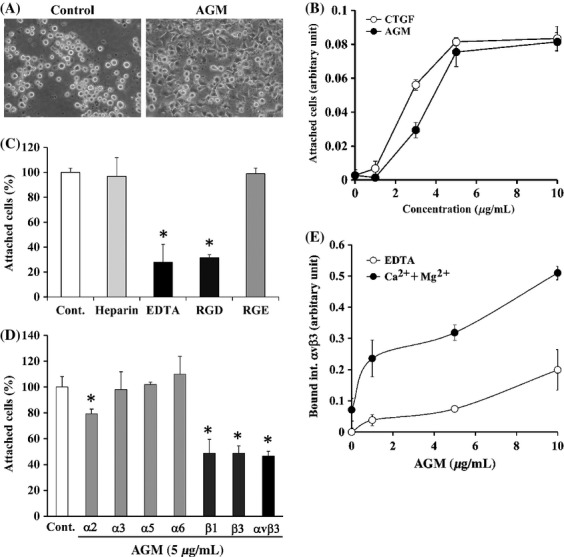
Effects of AGM on adhesion of human vascular endothelial cells. (A) HUVECs suspended in serum-free basal medium were incubated on plates precoated with PBS alone (Control) or 10 *μ*g/mL AGM, and their phase-contrast micrographs were taken after for 2-h incubation. Original magnification, ×300. (B) Ninty-six-well ELISA plates were precoated with the indicated concentrations of AGM (closed circles) or CTGF (open circles). HUVECs were incubated for 2 h on the coated plates. The relative number of adherent cells was measured as described in Materials and Methods. Each point represents the mean ± SD of the numbers of cells in triplicate wells. (C) Effects of heparin, EDTA, and RGD/RGE peptides on attachment of HUVECs to AGM. HUVECs were incubated for 2 h in the absence (Control) or presence of 100 *μ*g/mL heparin, 10 mmol/L EDTA, 1 mmol/L RGD, or 1 mmol/L RGE on the plates precoated with 5 *μ*g/mL AGM. The cells attached to the plates were measured as described above. *P < 0.01. (D) Effects of function-blocking anti-integrin antibodies on cell adhesion to AGM. HUVECs suspended in serum-free basal medium were incubated with control mouse IgG (Control) or with the indicated anti-integrin antibodies (20 *μ*g/mL IgG) on the AGM-coated plates. The relative number of adherent cells in the presence of control mouse IgG was taken as 100%. Each bar represents the mean ± SD of the triplicate assays. *P < 0.01. (E) Binding of recombinant integrin *α*v*β*3 to purified AGM. Indicated concentrations of purified AGM was coated on ELISA plates, and recombinant integrin *α*vß3 was allowed to bind to the AGM-coated plates in the presence of 10 mmol/L EDTA (open circles) or 10 mmol/L MgSO_4_ plus 1.6 mmol/L CaCl_2_ (closed circles). Integrin *α*v*β*3 bound to the plates was quantified by ELISA using the anti-integrin-ß3 antibody. The amounts of integrin *α*v*β*3 bound to AGM-free wells in the presence of 10 mmol/L EDTA was taken as nonspecific binding and subtracted as the background. The results shown are the means of triplicate assays. Other experimental conditions are described in the Materials and Methods.

To elucidate possible functions of AGM in cancer vasculature, we examined effects of purified AGM on some activities of human umbilical vein endothelial cells (HUVECs) in vitro. In the cell growth assay, purified AGM showed no growth effect on HUVECs in either presence or absence of VEGF (data not shown). AGM also did not support migration of HUVECs and was rather inhibitory in the two-well chamber assay (data not shown). However, when HUVECs were incubated on culture plates which had been precoated with AGM, they attached and spread on the coated plates but not on a PBS-treated plate (Fig. 4A). When the cell adhesion activity of AGM was compared at varied concentrations with that of connective tissue growth factor (CTGF), which is also a member of the IGFBP superfamily and known to have cell adhesion activity, both proteins showed similar activity (Fig. 4B) 37.

We previously reported that AGM weakly supports adhesion of fibroblasts through its heparin-binding site 38. In this study, however, the cell adhesion activity of AGM to fibroblasts appeared far lower than that to vascular endothelial cells (data not shown). In contrast with the case of fibroblasts, EDTA, but not heparin, effectively blocked the adhesion of endothelial cells to AGM (Fig. 4C). Although AGM does not contain the RGD sequence, RGD peptide, but not RGE, significantly blocked the cell adhesion to AGM (Fig. 4C). These results implied that integrins might mediate the cell adhesion to AGM. When various function-blocking, anti-integrin antibodies were tested, antibodies to integrins *α*v*β*3, *β*1 and *β*3 clearly blocked the cell adhesion activity of AGM (Fig. 4D). Anti-integrin *α*2 antibody appeared to weakly block the activity, but antibodies against integrins *α*3, *α*5, and *α*6 did not have significant inhibitory effect. These results strongly suggested that integrin *α*v*β*3 is a major receptor for AGM in endothelial cells, and integrin *α*2*β*1 and some other *β*1 integrins including *α*v*β*1 might support the cell adhesion to AGM to some extent.

Moreover, we examined direct interaction of AGM with integrin *α*v*β*3 by ELISA. Recombinant integrin *α*v*β*3 bound to AGM dependently on the AGM concentrations, and this binding was effectively blocked by EDTA, confirming that AGM indeed interacted with integrin *α*v*β*3 (Fig. 4E). Unexpectedly, the RGD peptide did not block the AGM binding to the integrin as analyzed by ELISA (data not shown). This was apparently inconsistent with the data of Figure 4C, which showed that the RGD peptide blocked the cell adhesion activity of AGM. These data seem to imply that AGM binds to integrin *α*v*β*3 at a distinct site from its RGD-binding site but in a manner by which the cell adhesion signaling from AGM is blocked by the RGD binding in living cells.

### Vascular permeability

**Figure 5:**
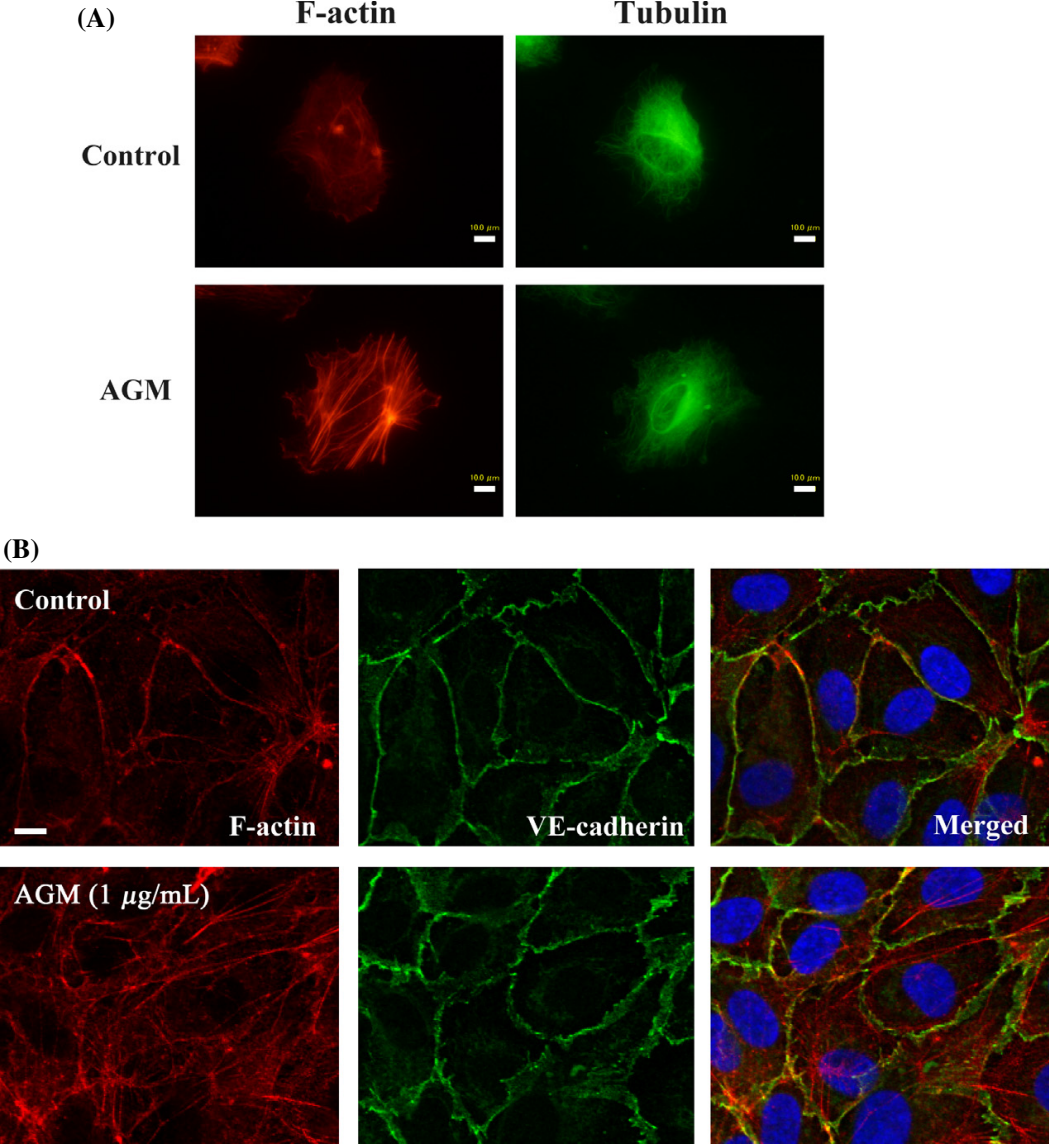
Effects of AGM on cytoskeletal structures and cell-cell junction. (A) Effect of AGM on cytoskeleton of HUVECs in sparse culture. A sparse culture of HUVECs in 1% serum-containing medium was incubated with 5 *μ*g/mL AGM for 2 days. F-actin was stained with rhodamine phaloidine (red), and microtubules with anti-*α*-tubulin antibody and FITC-labeled second antibody (green). Scale bars, 10 *μ*m. (B) Effect of AGM on localization of F-actin and VE-cadherin. A confluent culture of HUVECs were grown to confluence on fibronectin-coated 8-well Lab-Tek chamber slides (Thermo Scientific, Rochester, NY) and incubated in serum-free basal medium with 1 *μ*g/mL AGM for 3 h. F-actin (red) and VE-cadherin (green) were stained as above. Scale bars, 10 *μ*m.

**Figure 6:**
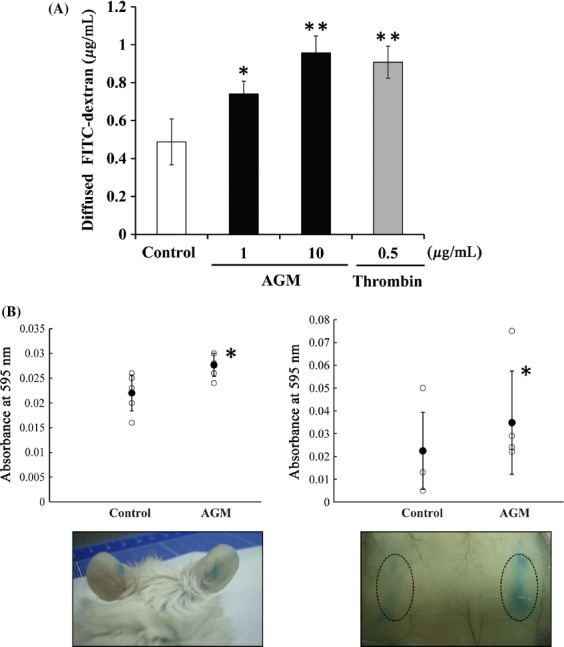
Stimulation of endothelial cell permeability by AGM in vitro and in vivo. (A) In vitro permeability assay. The monolayer of HUVECs on culture inserts with 0.4-*μ*m pores were treated with PBS or a test sample for 18 h and then with FITC-dextran 40 for 30 min. Fluorescence intensity of FITC-dextran diffused into the lower chamber was measured for the fluorescence. Each point represents the mean ± SE of the triplicate assay. *P < 0.05; **P < 0.01. (B) Miles vascular permeability assay in vivo. After Evans blue dye injection, AGM and PBS as control were injected intradermally into the right side and the left side of the ears (left panels) or the back skin (right panels) in the same mouse. Thirty min later, the leakage of Evans blue from blood vessels was measured. *P < 0.05. The lower panels indicate mice showing representative results in the two experiments. Other experimental conditions are described in Materials and Methods.

To investigate AGM-induced functional change of endothelial cells, cytoskeletal change of HUVECs was analyzed after the treatment with soluble AGM by the rhodamine-phalloidin staining. The AGM-treated cells showed prominent stress fibers as compared with nontreated cells (Fig. 5A). On the other hand, microtubule structures were unchanged or weakly attenuated by the AGM treatment. The AGM-induced stress fiber formation was also found even in confluent culture of HUVECs, resulting in disturbance of VE-cadherin-mediated cell-cell junction (Fig. 5B). These results gave rise to the possibility that AGM might affect the barrier function of endothelial cell sheet. In the two-well chamber assay, AGM increased permeability of HUVEC monolayer (Fig. 6A). Furthermore, Miles assay with mice showed that AGM significantly increased vascular permeability in vivo (Fig. 6B).

## Discussion

The present immunohistochemical analysis of breast cancers clearly showed that AGM was highly expressed in cancer-associated blood vessels regardless of noninvasive or invasive character. In DCIS, microvessels surrounding tumor islands showed characteristic AGM staining, while AGM was intensely detected in diffuse capillaries of invasive carcinomas. Furthermore, elevated expression of AGM was found in cancer-associated fibroblasts (CAFs), and this expression was correlated with macrophage infiltration as detected by CD68 antibody and with cancer invasiveness. Our recent study has shown that AGM is upregulated by TGF-*β* in normal fibroblasts and contributes to their activation and connective tissue formation in cancer tissues 21. On the other hand, the present study revealed that AGM was strongly induced in vascular endothelial cells by VEGF but not by TGF-*β*. Inflammatory cytokines, IL-6, IL-8, and TNF-*α*, weakly stimulated the AGM expression in endothelial cells. Thus, it is expected that AGM is induced in microvessles in a close vicinity of cancer cells primarily by VEGF expressed in cancer cells and endothelial cells themselves, though macrophage-derived inflammatory cytokines may also act as AGM inducers. Although our and a few other studies have shown elevated expression of AGM/IGFBP-rP1/IGFBP7 in the vasculature of some types of cancer tissues 15,16,21,39, others have reported opposite results 22–24. Burger et al. 22. reported that in the human breast, AGM/Mac25 expression was high in epithelial cells of normal lobules and ducts, moderate to weak in hyperplastic and DCIS cells and absent in invasive carcinomas. They found no expression of AGM in blood vessels of breast carcinomas. In our present study, we could not detect any AGM-positive signals in normal breast epithelial cells. This discrepancy may be derived from the difference in the antibodies used in the two studies. The two antibodies may recognize different epitopes of AGM or different antigens. We confirmed that a goat polyclonal antibody to IGFBP-rP1 (R&D) showed essentially the same immunostaining patterns as those obtained from the monoclonal antibody (clone 88; data not shown).

Blood vessels in cancer tissues are structurally and functionally distinct from those in normal tissues 5. It has been accepted that the vascular system in tumor tissues is irregular, unstable, and leaky compared to that of normal vessels 5,6. These structural and functional changes of blood vessels are thought to have significant effects on tumor growth and metastasis. Recently we found that a novel form of laminin, laminin-3B11, is present in the vascular basement membranes of various normal tissues, but its expression is lost in cancer tissues 32. Both AGM and laminin-3B11 are cell adhesion ligands assembled into the basement membrane structure, but their expression patterns are completely opposite as shown in this and previous studies. It is expected that the abnormal deposition of AGM, as well as the loss of laminin-3B11, in the vascular basement membrane of cancer tissues could give significant effects on the structure, permeability, and other functions of blood vessels. Recently, Hooper et al. 16. reported that in a zebrafish model AGM and VEGF synergistically regulate angiogenesis. They also showed that AGM is specifically expressed in the vasculature of developing embryos and adult organs of mice and upregulated in tumor tissues. Pen et al. 39. also identified AGM/IGFBP7 as a molecular marker that was most prominently upregulated in glioblastoma vessels. In addition, they found that AGM stimulates capillary-like tube formation of endothelia cells in Matrigel 40. One group has reported that AGM is expressed in high endothelial cells of mouse lymphoid tissues and interacts with mouse recombinant VEGF and some chemokines 36,41. In this study, we could not see significant affinity of AGM for VEGF in a similar assay with human proteins (data not shown). However, we have obtained data showing that some of AGM activities are greatly modulated by proteolysis 18 and glycosylation (E. Komiya and K. Miyazaki, unpublished data). It is also noted that CCN families such as CCN1 (CHR61) and CCN-2 (CTGF), which belong to the IGFBP superfamily, have binding sites for both VEGF and various integrins including integrin *α*v*β*3 42. Considering these facts, our results cannot exclude the possible direct interaction between AGM and VEGF.

Our present study first demonstrated that AGM had significant cell adhesion activity toward vascular endothelial cells through binding to integrin *α*v*β*3 and stimulated vascular permeability. The interaction of AGM with integrin *α*v*β*3 induced prominent stress fiber formation in endothelial cells and partially disrupted the VE-cadherin-mediated cell-cell adhesion system. These effects seem to cause the loss of barrier function of the endothelial cell sheet and the increase of vascular permeability. Integrin *α*v*β*3 is induced by VEGF in endothelial cells 43 and is an excellent biomarker of tumor angiogenesis 44. Although integrin *α*v*β*3 has both proangiogenic and antiangiogenic activities depending on experimental conditions 45,46, it has long been thought to be an important target for anticancer treatment. For example, Cilengitide, a cyclic RGD peptide, displays antiangiogenic effects both in vitro and in vivo 47 and has entered phase III clinical trials 35. Interestingly, this integrin *α*v*β*3 antagonist activates the integrin and blocks VE-candherin function, leading to the enhancement of endothelial monolayer permeability 48. Integrin *α*v*β*3 interacts with various ligands including vitronectin, fibronectin, fibrinogen, von Willebrand factor and osteopontin, many of which contain the RGD sequence. It has been reported that CTGF/CCN2, a non-RGD type ligand for integrin *α*v*β*3, promotes endothelial cell adhesion, migration and survival through the interaction with the integrin and promotes angiogensis in vivo 37. Osteopontin is a RGD-containing ligand of integrin *α*v*β*3, induced by VEGF in endothelial cells and promotes endothelial cell adhesion and migration through integrin *α*v*β*3 43. In the present study, we identified AGM as a non-RGD type novel ligand for integrin *α*v*β*3 in endothelial cells. Like CTGF and osteopontin, AGM is an extracellular protein that modulates cellular functions. However, AGM appears to play a different role in cancer vasculature from CTGF and osteopontin, because AGM did not support endothelial cell migration despite its cell adhesion activity. In our immunohistochemical analysis, CTGF was expressed in cancer cells but not vasculature of human breast cancers (data not shown).

AGM was initially identified as a tumor-derived cell adhesion factor (TAF) 12. AGM supports weak adhesion of fibroblasts by binding to syndecan-1 on the cell surface through its heparin-binding sequence 15,38. However, the cell adhesion activity of AGM was much stronger for endothelial cells than fibroblasts. We confirmed that the integrin *α*v*β*3 level of normal human fibroblasts was negligible compared to that of HUVECs (data not shown). Therefore, the differential cell adhesion activity of AGM between fibroblasts and endothelial cells seems to depend on the difference in their integrin *α*v*β*3 levels. Our results showed the direct interaction between purified AGM and a purified, soluble form of integrin *α*v*β*3. On the other hand, it is known that syndecan-1 directly interacts with and activates integrin *α*v*β*3 49. Therefore, it seems possible that interaction of AGM with syndecan-1 may also be involved in the endothelial cell adhesion via integrin *α*v*β*3. The most important feature of AGM is its prominent accumulation in cancer vasculature. Like integrin *α*v*β*3, AGM was found to be upregulated in endothelial cells by VEGF. Indeed, both AGM and integrin *α*v*β*3 were highly detected and colocalized in cancer-associated blood vessels. AGM is known to bind the basement membrane protein type IV collagen with high affinity 15. In the present study, we found that AGM indirectly bound to IV collagen via some heparan sulfate proteoglycans associated with this collagen (data not shown). It is expected that the increase of AGM level in the vascular basement membrane changes the cell adhesion ligands for endothelial cells from type IV collagen and laminins to AGM.

VE-cadherin and other proteins in the adherence junction play critical roles in the regulation of intercellular junction and vascular permeability. VEGF and other permeability factors affect the functions of these junctional proteins 50. Our results suggest that the binding of AGM to integrin *α*v*β*3 on endothelial cells activates this integrin and induces prominent actin stress fiber formation. This cytoskeletal change is thought to destabilize the VE-cadherin-mediated adherence junction and thereby increase vascular permeability. VEGF is well known to be a potent endothelial permeability factor. VEGF increases the production of reactive oxygen species (ROS), which causes tyrosine phosphorylation of VE-cadherin and *β*-catenin, leading to the impairment of vascular barrier function 51. It is expected that AGM acts as a cell adhesion ligand after sufficient accumulation in the endothelial basement membrane, while the growth factor VEGF rapidly acts on the cells at very low concentrations. VEGF-induced AGM seems to play a role in the long-term effect on vascular permeability in tumor microenvironment. Thus, VEGF is likely to have both the short-term stimulatory effect and the AGM-mediated long-term effect on vascular permeability. Moreover, it is noted that AGM has been identified as a prostacyclin-stimulating factor, which stimulates the production of the potent vasodilator PGI_2_ in fibroblasts 13. As an alternative mechanism, AGM-induced PGI_2_ may also contribute to the enhanced vascular permeability in the cancer vasculature.

The partial loss of endothelial cell junction is not only a trigger of hyper vascular permeability but also an important initial step of tumor angiogenesis and metastasis. AGM is a good molecular marker for activated blood vessels in human cancers and may be a therapeutic target for cancer treatment.
